# An overview of dysphagia rehabilitation for stroke patients

**DOI:** 10.1590/0004-282X-ANP-2021-0073

**Published:** 2022-01-31

**Authors:** Klayne Cunha Matos, Vanessa Fernandes de Oliveira, Paula Luanna Carvalho de Oliveira, Pedro Braga

**Affiliations:** 1 Hospital Geral de Fortaleza, Serviço de Fonoaudiologia, Fortaleza CE, Brazil. Hospital Geral de Fortaleza Serviço de Fonoaudiologia Fortaleza CE Brazil; 2 Universidade Federal do Ceará, Faculdade de Medicina, Fortaleza CE, Brazil. Universidade Federal do Ceará Faculdade de Medicina Fortaleza CE Brazil; 3 Universidade Estadual do Ceará, Centro de Ciências da Saúde, Fortaleza CE, Brazil. Universidade Estadual do Ceará Centro de Ciências da Saúde Fortaleza CE Brazil; 4 Universidade Federal do Ceará, Departamento de Clínica Médica, Fortaleza CE, Brazil. Universidade Federal do Ceará Departamento de Clínica Médica Fortaleza CE Brazil

**Keywords:** Deglutition Disorders, Stroke, Speech Therapy, Rehabilitation, Transtornos de Deglutição, Acidente Vascular Cerebral, Fonoterapia, Reabilitação

## Abstract

**Background:**

Dysphagia is characterized by difficulty in the swallowing pattern at any stage of this neuromuscular process. It is a frequent symptom after stroke.

**Objective:**

This study aimed to investigate the most commonly used phonoaudiological interventions as therapy for the treatment of swallowing disorders in patients with dysphagia after stroke.

**Methods:**

We performed a review of studies indexed in MEDLINE-PubMed, LILACS, Cochrane, and Clinical trials.gov focusing on speech-language interventions for adult dysphagic patients after stroke between January 2008 and January 2021.

**Results:**

Thirty-six articles of clinical trials were selected. Eleven different types of therapies have been studied. Studies on the efficacy of therapeutic interventions for the rehabilitation of adult patients with dysphagia after stroke are still scarce. Most techniques are combined with conventional therapy, so the effectiveness of the other techniques alone cannot be assessed.

**Conclusions:**

Therapeutic interventions should be selected in accordance with the possibilities and limitations of the patients, and especially with the findings of the clinical evaluation and with its objective.

## RESUMO

**Antecedentes:**

A disfagia é caracterizada como uma dificuldade no padrão de deglutição em qualquer fase desse processo neuromuscular. É um sintoma frequente após o Acidente Vascular Cerebral.

**Objetivos:**

O objetivo deste estudo foi investigar as intervenções fonoaudiológicas mais utilizadas como terapia para o tratamento dos distúrbios da deglutição em pacientes com disfagia pós AVC.

**Métodos:**

Realizamos uma revisão dos estudos indexados no MEDLINE-PubMed, LILACS, Cochrane e Clinical trials.gov com foco nas intervenções fonoaudiológicas em pacientes adultos e disfágicos após AVC entre janeiro de 2008 e janeiro de 2021.

**Resultados:**

Foram selecionados trinta e seis artigos de ensaios clínicos e estudados onze tipos de terapia. Os estudos sobre a eficácia de intervenções terapêuticas para a reabilitação destes pacientes adultos ainda são restritos. A maioria das técnicas é aplicada em combinação com a terapia convencional, tornando inconclusiva a medição da eficácia de outras técnicas isoladamente.

**Conclusões:**

As intervenções terapêuticas devem ser escolhidas de acordo com as possibilidades e limitações dos pacientes e, principalmente, com os achados da avaliação clínica e seu objetivo.

## INTRODUCTION

Dysphagia is characterized by difficulty or discomfort in swallowing. The swallowing process ensures the transit of food and saliva from the oral cavity to the stomach providing metabolic balance, nutrition, and hydration to the human body^1^. Stroke is the main cause of neurogenic dysphagia^2^. Depending on the affected area and the extent, stroke can lead to damage to the neurological control areas of swallowing, which may cause dysphagic disorders^1^. 

Dysphagia after stroke carries the risk of aspiration pneumonia, malnutrition, dehydration, and death^3^. Dysphagia is a major complicating factor in the rehabilitation of such patients or of neurological patients in general. If associated with other risk symptoms such as drowsiness, disorientation or incontinence it may indicate an even worse prognosis^4^^,^^5^. Recent studies indicate an average frequency of dysphagia of 50% after stroke. It causes a large increase in hospital costs per patient. Patients with stroke who develop dysphagia have a longer length of hospital stay when compared to not dysphagic patients, requiring additional care for enteral diets and are more likely to need home care after discharge^6^. 

Considering the need for early intervention for dysphagia in post-stroke patients to improve their quality of life and reduce sequelae, complications, hospital costs and hospitalization time, it is necessary to find effective treatment interventions. The objective of this study was to investigate the most commonly used phonoaudiological interventions as therapy for swallowing disorders in patients with dysphagia after stroke. In this review, we provided the most relevant information of dysphagia treatment after stroke published in recent years. 

## METHODS

### Eligibility criteria 

Studies were selected according to predefined inclusion and exclusion criteria. “English-language full-text articles on clinical and controlled trials indexed in the previously selected electronic databases with adult populations presenting with oropharyngeal dysphagia as a symptom after stroke” was defined as inclusion criteria. Studies involving (1) pediatric population, (2) analysis of the application of evaluation protocols, (3) studies aiming at decannulation of tracheotomized patients, (4) studies involving esophageal dysphagia, and (5) studies in which the primary outcome was not related to the degree of dysphagia and improvement of the swallowing pattern were excluded. 

### Review question

The guiding question for the research was: "What interventions are reported as effective treatments for the rehabilitation of adult patients with dysphagia after stroke?".

### Search strategy, study selection and data extraction

A review of articles published between January 2008 and January 2021 in indexed scientific journals was carried out in the following electronic databases: MEDLINE-PubMed, LILACS, Cochrane, and Clinical Trials.gov. The selection of descriptors was performed through consultation in a Brazilian platform for descriptors in health sciences (DeCS -Descritores de Ciências em Saúde). The selected English descriptors were: "dysphagia" AND "therapy" AND "stroke". 

The articles were selected based on the screening of titles or abstracts. However, when title, keywords, and abstract did not have sufficient information to determine the inclusion according to the established criteria, a full-text review was conducted. After that, all the remaining papers were fully read, evaluated, and cataloged. All steps in this study were performed independently by two researchers following the protocol described above. Individual results were assessed and compared, and a consensus was reached through discussion. 

The following data were extracted after the assessment of full text of all selected articles: country, number of patients, study design, outcome measures, types of intervention groups, intervention time, summary of the results, and conclusions. These data were then compiled into a standard table. 

## RESULTS

**Figure 1.**
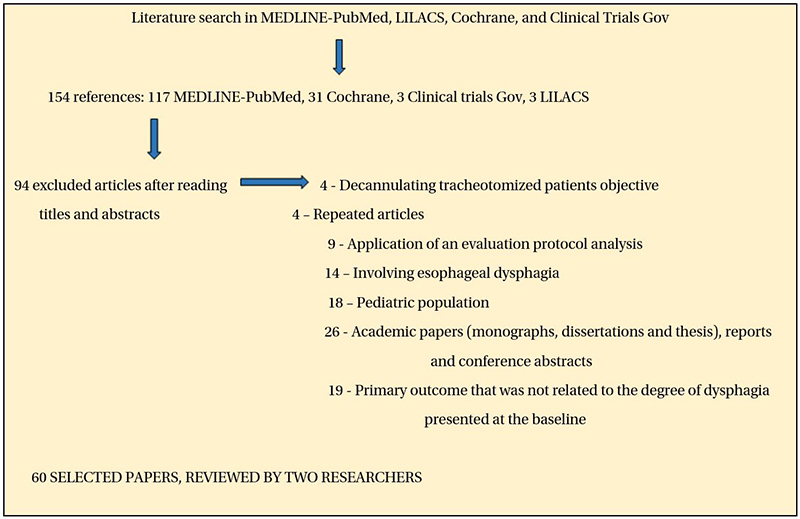
Flow chart of the search strategy.

 The search identified 154 references (117 in MEDLINE-Pubmed, 31 in Cochrane, 3 in Clinical trials, and 3 in LILACS) of which 90 were excluded based on the title and 4 were duplicates. Therefore, 60 studies were selected for inclusion according to their titles and abstracts, of which 38 clinical trials that met the inclusion criteria were included in this review. A flow diagram showing the study selection process is presented in Figure 1.

All included studies evaluated patients diagnosed with stroke. A total of 12 different therapies have been studied with a variety of study designs: [1] electrical stimulation (n = 14; 36.8% of the total)^7^^-^^20^, [2] transcranial magnetic stimulation techniques (n = 3; 7.9%)^21^^-^^23^, [3] active pharyngeal electrostimulation (n = 3; 7.9%)^24^^-^^26^, [4] exercises with Mendelsohn maneuver (n = 2; 5.3%)^27^^,^^28^, [5] transcranial direct current stimulation (n = 3; 7.9%)^29^^-^^31^, [6] CTAR exercise (n = 5; 13.6%)^32^^-^^36^, [7] Shaker exercise (n = 2; 5.3%)^33^^,^^37^, [8] acupuncture (n = 3; 7.9%)^38^^-^^40^, [9] resistance to tongue pressure (n = 2; 5.3%)^41^^,^^42^, [10] modified jaw opening exercise (n = 1; 2.6%)^43^ and [11] cervical isometric exercises (n = 1; 2.6%)^44^.

Even among studies focusing on the same type of therapy a wide variety of outcome measures were found to assessing dysphagia. The sample sizes varied from 4^22^ to 250^40^. More than half of the studies were from Asia, but some were from Europe and North America. 

The following sections present a summary of the articles’ results by type of interventions.

### Electrotherapy (NMES) 

Electrotherapy is a technique that can be used with motor stimuli, sensorial stimuli, or both. In addition, depending on the muscular function affected in the swallowing process and the degree of this change, variations in intensity, electric current pulse duration, electrodes number and position are applied. Despite all these factors, there is still little scientific evidence of the effectiveness of electrotherapy in improving the swallowing pattern in oropharyngeal dysphagia, especially when this technique is associated with conventional exercise therapy.

The Neuromuscular Electrical Stimulation (NMES) technique combined with Endoscopic Evaluation of Swallowing (EES) and traditional swallowing rehabilitation improved the swallowing quality in a study involving thirty-two patients with moderate to severe post-stroke dysphagia. In addition, patient satisfaction was high and there were no serious adverse events. Thus, the implementation of this promising combination in clinical practice was recommended^7^. Similarly, the effects of applying sensory-level electrical stimulation (SES) on masseter muscles in patients with acute stroke were evaluated in another study^8^. Applying SES (based on its oral and pharyngeal functions) on masseter muscles and using SES to generate cortical reorganization was effective in treating dysphagia in stroke patients. 

In another study^17^, selected patients were randomly assigned to a VitalStim electrotherapy group, a conventional swallowing therapy group, and electrotherapy plus conventional therapy group. The results suggested that VitalStim combined with conventional therapy is capable of improving dysphagia after stroke. Xia et al.^18^ also evaluated the effects of VitalStim in their patients and confirmed its effectiveness. A limitation of this study, however, was the absence of a placebo stimulation group.

The combined application of electrical stimulation and conventional therapy in patients with acute oropharyngeal dysphagia secondary to acquired brain injury^9^ resulted in better outcomes than when conventional therapy with placebo stimulation was adopted. A study conducted by Rofes et al.^10^ evaluated and compared the efficacy and safety of a 10-day surface electrical stimulation (e-STIM) treatment in sensory and motor intensities in patients with chronic oropharyngeal dysphagia after chronic stroke. This study showed that e-STIM is a safe and effective therapy to improve swallowing. However, further investigation involving a control group, a larger number of patients, a prolonged follow-up, and the effect on clinical outcomes is needed to confirm the clinical utility of this therapy.

Konecny and Elfmark (2018) showed that after four weeks of standard therapy with suprahyoid muscles electrical stimulation, the duration of oral and pharyngeal transit time was statistically improved^11^. In another study, electrical stimulation associated with conventional therapy was performed. Electrodes were placed adjacent to the suprahyoid and upper and lower parts of the thyroid, in the geniohyoid region, and in the mylohyoid region. When compared to conventional therapy, a significant improvement was noted. However, the position of electrodes did not generate significant differences^12^.

Another study investigated the effects of forced swallowing combined to neuromuscular electrical stimulation on the hyoid bone movement and on the swallowing function in stroke patients^13^. The experimental group showed an increase in the anterior and superior movement of the hyoid bone and an improvement in the pharyngeal phase swallowing function. The neuromuscular electrical stimulation combined with thermal-tactile stimulation were found to be a better treatment for patients with deglutition disorders after stroke than isolated thermal-tactile stimulation therapy^14^. 

Electrical stimulation was performed by Park et al.^15^ using two sets of electrodes placed in the segmentation area of the infrahyoid and sternum-hyoid muscles. When using sensorial EE with forced swallowing, no significant improve was observed in any of the evaluated parameters. However, the studied methodology can only be applied to selected patients, as many patients with dysphagia fail to elevate the hyolaryngeal complex during motor electrical stimulation.

The effects of NMES associated with electromyographic biofeedback (EMG-BF) were also investigated^19^. EMG-BF is known to be an effective therapy for stroke rehabilitation. In this pilot study, all subjects received NMES combined with EMG-BF in the suprahyoid area. The results demonstrated that NMES combined with EMGBF had the potential to improve oropharyngeal swallowing in stroke patients with dysphagia.

In a more recent study, the effects of neuromuscular transcutaneous electrical stimulation (NMES) in 33 patients affected by dysphagia after sub-acute stroke were evaluated. Both groups showed improvements^16^. Another recent study analyzed the McNeill's dysphagia therapy (MDTP) with NMES for the treatment of post-stroke dysphagia^20^. MDTP showed a greater positive change than the NMES group, including increased oral intake and improved functional outcome three months after the stroke. These data support the inclusion of intense short-term behavioral interventions for an efficient allocation of resources for acute stroke rehabilitation.

Studies that used electrotherapy as a therapeutic approach are detailed in Table 1.

**Table 1.** Clinical studies using electrotherapy for patients after stroke or TBI.

References	Country	Patients	Diagnosis	Study design	Outcomes	Intervention	Intervention time	Results	Follow-up
Terré,R.; Mearin, F., 2014^9^	Spain	20	STROKE and TBI	Randomized; controlled; prospective	VFSS; FOIS	GI-EE and TC GC -EE placebo eTC	20 sessions/60 min-5 times a week	FOIS increased 4.9 points (GI); 3.1 points (GC).	1, 3 months
Sun, S.F et al., 2013^7^	Taiwan	29	STROKE	Clinic; prospective	FOIS; Dysphagia scale	EE e TC separately	NMES and TC 12 sessions/60 min -3 times a week	FOIS and dysphagia scale improved after NMES, for 6 months and 2 years (p \ 0.001, each)	6 months, 2 years
Rofes, L. et al., 2013^10^	Spain	20	STROKE	Randomized; double-blind	VFSS (PAS)	Motor EE group Sensory EE group	10 sessions/ 30 min -5 times a week	Sensory and motor EE reduced the insecure deglutition number in (p < 0.001), and (p = 0.002)	No follow-up
Park, J. S. et al., 2016^13^	Korea	50	STROKE	Randomized; Controlled; Single-blind	VFSS (PAS e VDS)	GI -EE and forced swallowing exercise GC -EE placebo and forced swallowing exercise	30 sessions/30 min -5 times a week	GI increased oral and pharyngeal phase in VDS (P < 0.00, P = 002 and P < 0.00), and PAS (P < 0.00).	No follow-up
Park, J. W. et al., 2012^15^	Korea	20	STROKE	Randomized; Controlled; Double-blind	VFSS (PAS; UES)	GI -Motor EE and forced swallowing GC-Sensory EE and forced deglutition	12 sessions/20 min -3 times a week	GI increased vertical larynx movement (p\0.05).	No follow-up
Konecny, P.; Elfmark, M., 2018^11^	Czech Republic	108	STROKE	Randomized; Controlled; Prospective	VFSS	GI -Motor EE and TC GC -TC	20 sessions/20 min -5 times a week	The difference in the oral and pharynx transit time after therapy between the GI and the GC (P = 0.01 e P= 0.009)	No follow-up
Lim, K. B. et al., 2009^14^	Korea	28	STROKE	Randomized; Controlled	VFSS	GI -EE muscular and tactile-thermic stimulation GC -Tactile-thermic stimulation	20 sessions/60 min -5 times a week	GI with higher scores in PAS; 2 in semi-solid (p < 0.05) and 2.5 in liquids (p < 0.05)	No follow-up
Meng, P. et al., 2017^12^	China	30	STROKE	Randomized	VFSS; DOSS	GA -EE with electrodes along the suprahyoid and along with the superior and inferior thyroid parts and TC GB -EE with 1 pair of electrodes in the geniohyoid region and 1 pair in the mylohyoid region and TC GC -TC	10 sessions/30 min -5 times a week	Improvement in DOSS in groups A and B (P<0.005) in relation to GC.	No follow-up
Xia, W. et al.,2011^18^	China	120	STROKE	Randomized; prospective	SSA; VFSS	G1 -Conventional therapy G2 -EE G3 -Conventional therapy and EE	40 sessions/30 min -2 times a day, 5 times a week for 4 weeks	SSA, VFSS increased more in G3 than in G1 and G2 (P < 0.01).	No follow-up
LI, L et al., 2015^17^	China	135	STROKE	Randomized; controlled	SSA	G1 -EE G2 -TC G3 -EE and TC	20 sessions/60 min -5 times per week for 4 weeks	SSA improved in G3 (P <0.01)	4 weeks
Ploumis, A. et al., 2018^44^	Greece	70	STROKE	Randomized, controlled, prospective.	VFSS; PAS	GI -Cervical exercises and conventional therapy GC -Conventional therapy	30 min daily sessions/12 weeks	Improved swallowing (P < 0.05) and PAS (P < 0.001)	No follow-up
Umay, E. et al., 2017^8^	India	98	STROKE	Randomized; controlled	MASA; SSA	GI -Sensory EE and TC GC -Sensory EE placebo and TC	20 sessions/60 min -5 times a week for 4 weeks	All parameters improved in G1 (P <0.025).	No follow-up
Park, S.J. et al., 2019^34^	Korea	10	STROKE	Clinical; prospective	VFS; PAS	NMES and EMG-BF	20 sessions/30 min -5 times a week	Significant differences between oral (P = 0.015) and pharyngeal (P = 0.016) VFS. Improved PAS (P = 0.031).	No follow-up
Carnaby, G.D et al., 2020^20^	USA	53	STROKE	Randomized; controlled; double-blind	FOIS, MASA	G1 -TC and EENM G2 -TC and EENM placebo G3 -TC	15 sessions/60 min -3 weeks	MASA was different among groups different (p ≤ 0.0001) G2 had the best FOIS result (p≤0.0001).	3 months

DOSS: Dysphagia Outcome and Severity Scale; EE: Electrical Stimulation; NMES: Neuromuscular Electrical Stimulation ; FOIS: Functional Oral Intake Scale; GA: Group A; GB: Group B; GC: Control group; GI: Intervention group; MASA: Mann Assessment of Swallowing Ability; PAS: Penetration-Aspiration Scale; SSA: Standardized Swallowing Assessment; TC: Conventional therapy; TBI: Traumatic Brain Injury; VDS: videofluoroscopic dysphagia scale; UES: Upper esophageal sphincter; VFSS: Video Fluoroscopic Swallowing Study.

### Neuromodulation

The nervous system has the ability to modulate and modify itself in response to external stimuli. The term neuromodulation has been used to describe procedures in which electrical stimulation is applied directly to structures of the nervous system for therapeutic purposes. A summary of these approaches is shown in Table 2.

**Table 2.** Intervention clinical studies using neuromodulation for stroke patients.

Reference	Country	Patients	Diagnosis	Study design	Outcomes	Intervention	Intervention time	Results	Follow-up
Hyun, Y. et al., 2017^29^	Korea	26	Stroke	Randomized; multicenter controlled; prospective; double-blind.	DOSS	GI -ETCC and TC. GC -Placebo ETCC and TC	10 sessions/20 min -5 times a week	DOSS -Significant improvement (0.62 points on GI)	No follow-up
Cheng, I. K. Y. et al., 2017^21^	China	15	Stroke	Randomized; controlled; double-blind.	VFSS; IOPI	GI -Active EMTr GC -Simulated EMTr	10 applications -5 times a week	No significant results	2, 6 and 12 months
Cheng, I. K. Y. et al., 2014^22^	China	4	Stroke	Randomized; controlled.	VFSS; IOPI	GI -Active EMTr/ Tongue motor cortex stimulation GC -Simulated EMTr	10 sessions/30 min -5 times a week	No significant deglutition improvement on GI	1 week, 1 month
Du, J. et al., 2016^23^	China	40	Ischemic stroke	Randomized; controlled; double-blind.	SSA	G1 -High-frequency EMTr (3Hz) G2 -Low-frequency EMTr (1Hz) G3 -Simulated EMTr	5 sessions	Better G1 and G2 dysphagia improvement after 5 days compared to the other group, remaining for 5 months.	1, 2 and 3 months
Park, J.W. et al., 2013^15^	Korea	18	Stroke	Randomized; controlled; double-blind.	VFSS (PAS and VDS)	GI -Contralesional pharyngeal motor cortex EMTr 5Hz GC -Placebo (same conditions)	20 sessions/10 min	VDC and PAS improved significantly on the GI (P<0.005)	2 weeks
Shigematsu, T. et al., 2013^30^	Japan	20	Stroke	Prospective; double-blind.	DOSS	GI -TC and 1-mA ETCC (contralesional pharyngeal motor cortex) GC -Simulated ETCC and TC	10 sessions/20min -Once a day	1.4 points (P=0.006) improvement and after 1 month 2.8 points (P=0.004) improvement on the GI.	1 month
Krueger, S. S. et al., 2018^31^	Germany	59	Ischemic stroke	Randomized; double-blind.	FEDSS; SSA	Contralesional pharyngeal motor cortex ETCC group Placebo ETCC group	4 sessions/20min	Significant dysphagia improvement on the ETCC group when compared to the placebo group (P<0.0005)	No follow-up

DOSS: Dysphagia Outcome and Severity Scale; ETCC: TC: Conventional Therapy; GI: Intervention group GC: Control group; VFSS: Videofluoroscopy; IOPI: Iowa Oral Performance Instrument; EMTr: PAS: Penetration-Aspiration Scale; VDS: Functional Dysphagia Scale; SSA: Standardized Swallow Assessment; FEDSS: fiberoptic endoscopic dysphagia severity scale.

*Repetitive Transcranial Magnetic Stimulation*

Repetitive Transcranial Magnetic Stimulation (rTMS) has been proposed as an alternative treatment for dysphagia after stroke. It is a noninvasive technique that modulates brain activity using electromagnetic induction and thus induces physiological changes. An advantage of rTMS is that patients do not need to be actively engaged during treatment^21^.

One of the included studies indicated that 5 Hz rTMS applied over the tongue area of the motor cortex for 10 days was not effective in improving the swallowing function in patients with stroke and chronic dysphagia. However, given he small and unbalanced sizes of the groups in this study, the therapeutic effects of the protocol remain uncertain^21^. Another study also evaluated the therapeutic effect of 5 Hz high-frequency rTMS on the unaltered pharyngeal motor cortex in 4 post-stroke dysphagic patients. In disagreement with the previous study, the authors indicated that 5Hz high-frequency rTMS applied to the tongue region of the motor cortex may be beneficial for patients with dysphagia after hemispheric unilateral stroke and with dysfunction in the swallowing phase. Further investigations with larger samples are required to support the benefit of this protocol^22^. Finally, a study of 40 patients showed that the use of high frequency (3 Hz) and low frequency (1 Hz) rTMS improved dysphagia (after 5 days) more than the simulated group, with the effects remaining for at least 3 months after the intervention^23^.

*Transcranial direct current stimulation (tDCS)*

tDCS is a non-invasive brain stimulation method based on the principle of neuroplasticity. It provides a constant low-intensity electric current between the anode and the cathode applied to the scalp area associated with the segmentation of the cerebral cortex. In general, cathodic tDCS decreases cortical excitability and anodic tDCS increases cortical excitability^29^. Recently, noninvasive cortical stimulation has been used to improve neural plasticity and treat hemiplegia and aphasia. However, little is known about the possible effects of tDCS on swallowing function^30^, and few studies were conducted on the mater.

The association of the tDCS technique with conventional therapy was evaluated in patients with chronic post-stroke dysphagia. Although the result of this study shows that the bihemispheric anodic tDCS group did not have a statistically superior improvement compared with the control group, the detailed dysphagia outcome scale (using videofluoroscopy), patient symptom report, or patient and caregiver satisfaction may reflect the clinical improvement of dysphagia^29^. The study conducted by Shigematsu et al.^30^ investigated the effects of cerebral pharyngeal cortex noninvasive stimulation combined with intensive swallowing therapy on dysphagia recovery and found that the combined therapies effectively improve post-stroke dysphagia compared to isolated therapy.

Krueger et al.^31^ evaluated patients with acute and dysphagic stroke that received contralesional anode stimulation or placebo tDCS for 4 consecutive days. Applying objective instrumental diagnosis in parallel with functional neuroimaging, a greater improvement in the swallowing function was observed after tDCS compared with the placebo intervention. Thus, tDCS seems to be a safe and beneficial therapeutic option for patients with oropharyngeal dysphagia during the early stage of stroke.

### Pharyngeal electrical stimulation (PES)

In one of the included studies, PES interventions were performed at the bedside. The effects of PES on dysphagia in stroke patients remained inconclusive because the recruitment goal was smaller than predicted. Despite this, there is an indication for the use of this treatment considering some potentially favorable results, such as the observed improvement in the number of safe swallows. In addition, PES was well tolerated without any adverse effects^25^. In another study with the same objective, it was found that PES did not reduce radiological aspiration or clinical dysphagia^24^.

In addition, there are currently a wide variety of candidate genes that can be studied in the context of brain plasticity and response to PES. BDNF is the most abundant growth factor in the brain and is involved in long-term brain plasticity. It has attracted much interest and is considered a candidate for neurological and swallowing function recovery in patients treated with electrical stimulation of the pharynx^26^. The study conducted by Essa et al.^26^ aimed to test the possible influence of a single but common BDNF polymorphism on the functional recovery in a population with dysphagia after stroke. An association between the Val66Met BDNF allele and level of swallowing recovery was observed when pharyngeal stimulation was performed. On the other hand, the BDNF showed no correlation in the simulated group, suggesting that such genetic polymorphisms may be less relevant in natural recovery than in treatment-induced recovery.

A summary of studies using the pharynx electrostimulation technique is shown in Table 3.

**Table 3.** Interventional clinical studies using electrostimulation of the larynx, tongue pressure resistance exercise and precision training for patients after stroke.

References	Country	Patients (n)	Diagnosis	Study Design	Outcomes	Intervention	Intervention time	Results	Follow-up
Bath, P. M. et al., 2016^24^	United Kingdom	162	Stroke	Multicenter; Randomized; Controlled; Double-blind	VFSS (PAS)	GI -active PES GC -simulated PES	3 sessions/10 min	No GI improvements in relation to the GC	2,6 and 12 weeks
Vasant, D, H et al., 2016^25^	United Kingdom	36	Stroke	Multicenter; Randomized; Controlled	DSR	GI -active PES GC -simulated PES	3sessions/10 min	In relation to the simulated group, a probability ratio (OR)> 1 indicated a favorable outcome for the active group in DSR punctuations.	2 weeks and 3 months
Essa, H., 2017^26^	United Kingdom	38	Stroke	Randomized; Controlled; Double-blind	DRS	GI -active PES GC -simulated PES	3 sessions/10 min	In the GI, patients with the allele Met BDNF showed improvements in DERD after 3 months in relation to patients in the GC (P = 0.009)	2 weeks and 3 months
Moon, J. H et al., 2018^41^	Korea	16	Stroke	Randomized; Controlled	IOPI; MASA	GI -TPSAT in the morning and TC in the afternoon GC -TC	80 sessions/30 min -2 times a day, 5 times a week for 8 weeks	Anterior (P = 0.001) and posterior (P = 0.001) PMI improvement in the GI in relation to the GC; GI and GC MASA improvement (P = 0.012)	No follow-up
Kim, H. D. et al., 2016^35^	Korea	35	Stroke	Randomized; Controlled	IOPI; VFSS (VDS and PAS)	GI -TPRT and TC GC -TC	20 sessions -5 times a week	GI tongue strength improvement (anterior and posterior, p = 0.009, 0.015) and oral and pharynx phases punctuations improvement in VDS (p = 0.029, 0.007), but not in PAS (p = 0.471) in relation to the control group.	No follow-up

VFSS: Video Fluoroscopic Swallowing Study; PAS: Penetration-Aspiration Scale; DSR: Dysphagia Gravity Scale; DERD/DSRS: Dysphagia Severity Rating Scale; GI: Intervention Group; GC: Control Group; PES: Pharyngeal electric stimulation; BDNF: brain-derived neurotrophic factor; DRS: Dementia Rating Scale; PMI: maximum isometric pressure; VDS:videofluoroscopic dysphagia scale; TPRT: tongue to palate resistance training; IOPI: Iowa Oral Performance Instrument.

### Tongue pressure resistance exercise and precision training

Tongue function can affect both the oral and the pharyngeal stages of the swallowing process. Adequate tongue strength is vital for safe oropharyngeal swallowing. Table 3 has a summary of the studies on tongue pressure resistance exercises and precision training.

Kim et al.^42^ investigated the effect of tongue-pressure resistance training (TPRT) on tongue strength and oropharyngeal swallowing function in patients with stroke and dysphagia. The results showed that TPRT increased tongue muscle strength and improved swallowing function in patients with post-stroke dysphagia. This study also confirmed that TPRT improved the oral and pharyngeal phases of deglutition. Therefore, TPRT is recommended as an easy and simple rehabilitation strategy to improve swallowing in patients with dysphagia. However, these results do not reflect a pure TPRT effect, as this therapy was conducted in conjunction with conventional therapy^42^. Another study published the following year aimed to investigate the effects of tongue pressure strength and accuracy training (TPSAT) on tongue pressure strength and its ability to improve quality of life in patients with dysphagia after stroke. TPSAT consisted of an isometric exercise of anterior and posterior tongue strength and an isometric tongue precision exercise. TPSAT combined with traditional therapy improved outcomes compared to pre-intervention levels^41^.

### CTAR exercise

Recently, CTAR (Chin Tuck Against Resistance) exercise has been reported as a treatment for pharyngeal dysphagia. However, clinical evidence of its effect is still unclear. Park et al.^32^ investigated the effect of CTAR on the swallowing function in patients with dysphagia after subacute stroke and found that the exercise improved swallowing. 

Game-based CTAR was also proposed^34^. The experimental group performed game-based CTAR, while the control group performed traditional head lifting exercises. The LES 100 (Cybermedic Inc., Iksan in South Korea) consists of a tablet screen, a resistance bar, and a Bluetooth connector, and it implements a game-based exercise in which the chin is tucked down against a bar in order to strengthen suprahyoid muscles. The game-based CTAR not only has a similar effect on the swallowing function of patients with dysphagia as the lifting exercise, but is also a less rigorous, more enjoyable and interesting rehabilitation method.

Because the CTAR involves hand-holding a device, physically weak patients may find it difficult. A study investigated the effect of modified CTAR (mCTAR) in patients with post-stroke dysphagia^35^ and found that it reduced aspiration and improved nutritional levels of patients. I can thus be assumed that the mCTAR is beneficial for physically vulnerable patients with dysphagia who have limited hand strength and movement.

The aim of the study was to investigate the effect of jaw opening exercise (JOE) and hyoid bone movement compared to head lifting exercise, or Shaker exercise (HLE) in patients with dysphagia after stroke. The JOE/CTAR group performed an exercise using a resistance bar. The Shaker group performed traditional exercises. The total duration of the intervention was 6 weeks. The thickness of the digastric and mylohyoid muscles was measured by ultrasound. The CTAR and Shaker had similar effects in increasing the thickness of the suprahyoid muscle and improving the movement of the hyoid bone. However, CTAR required less perceived effort than Shaker^36^.

### Shaker exercise

The Shaker exercise (SE) has been considered a popular rehabilitation training for dysphagia^33^. This is an isometric and isotonic exercise based on the upward and forward movement of the larynx structures resulting from the traction of the thyroid, mylohyoid, and geniohyoid muscles and the anterior belly of the digastric muscle. First, patients perform 3 head raises for 60 s in a supinated position without movement; there is a 60 s pause between the elevations. Next, participants perform 30 repeated head raises in the supine position. Participants raise their head high enough to observe the toes without raising the shoulders^37^. 

Gao & Zhang^33^ compared the effects of Shaker exercises, CTAR and conventional exercises on dysphagia and psychological status. Traditional rehabilitation included tongue exercises such as tongue extension movement and mouth exercises such as mouth opening, teeth clicking, and voluntary swallowing. The main conclusion of this study was that the CTAR exercise has a similar effect on improving swallowing function as the Shaker exercise. However, the rehabilitation effect of CTAR exercises on dysphagia should be more explored in younger patients with stroke, since all patients assessed in this study were 60 years old or older. 

Choi et al.^37^ investigated the effects of the Shaker exercise on aspiration and oral diet level in stroke survivors with dysphagia. This study suggested that the SE is an effective exercise for swallowing function recovery in stroke survivors, reducing aspiration and improving oral diet level. As aspiration severity is closely related to the feeding tube and to the oral diet level, the results of this study indicate that performing SE can lead to tube withdrawal in stroke survivors with dysphagia. Some limitations, such as a relatively small sample, no follow-up after the intervention, and failure to observe long-term effects prevent the results of this work from being generalized. 

Important data from the articles about CTAR and SE are shown in Table 4.

**Table 4.** Intervention clinical studies using CTAR and Shake exercises for patients after stroke.

Reference	Country	Patients	Diagnosis	Study design	Outcomes	Intervention	Intervention time	Results	Follow-up
Gao, J.; Zhang, H.J., 2017^33^	China	90	Ischemic stroke	Clinical; Random	VFSS (PAS)	GC -TC Shaker -TC and Shaker exercise CTAR -TC and CTAR	42 sessions -3 times a day	A better swallowing improvement in the CTAR group when compared to the Shaker group	2, 4 and 6 weeks
Park, J. S. et al., 2018^32^	Korea	22	Stroke	Randomized; Controlled	VFSS (PAS; FDS)	GI -CTAR and TC GC -TC	20 sessions/30 min -5 times a week	Significant improvement in the PAS and FDS in the GI when compared to the GC	No follow-up
Choi, J. B. et al., 2017^37^	Korea	31	Stroke	Randomized; Controlled; Double-blind	VFSS (PAS); FOIS	GI -TC and Shaker exercise GC -TC	20 sessions/30 min -5 times a week	PAS and FOIS significantly improved the GI in relation to the GC	No follow-up
Park, J. S et al., 2019 ^34^	Korea	37	Stroke	Randomized; Controlled	VFSS (PAS); FOIS	GI -Game-based CTAR GC -CTAR	20 sessions/30min -5 times a week	There were no differences in improvement between groups	No follow-up
Kim, H. H.; Park, J. S, 2019^35^	Korea	30	Stroke	Clinical; Randomized	PAS; FOIS	GI -CTAR and TC GC -TC	30 sessions/30 min -5 times a week	GI had a significant improvement in PAS and FOIS (P <0.001, both)	No follow-up

VFSS: Videofluoroscopy; PAS: Penetration-Aspiration Scale; FDS: Functional Dysphagia Scale; GI: Intervention group; GC: Control group; TC: Conventional therapy; FOIS: Functional Oral Intake Scale; CTAR: Chin Tuck Against Resistance.

### Modified jaw opening exercise (MJOE)

The viability and effectiveness of a new method (modified jaw opening exercise -MJOE) for promoting anterior displacement of the hyoid bone during swallowing was studied. The MJOE differs from the conventional JOE, in which an upward vertical resistance is applied to the jaw while the mouth is closed with the tongue held in the swallowing tilting position to prevent mouth opening. In the MJOE, surface electrodes connected to the sternohyoid muscle in the mandibular midline were connected to the biofeedback equipment. The results showed that MJOE is feasible in elderly post-stroke patients, without adverse events and promotes anterior displacement of the hyoid bone during swallowing^43^.

### Mendelsohn maneuver 

The Mendelsohn maneuver, a voluntary prolongation of laryngeal elevation during swallowing, has been widely used as a compensatory strategy to improve the opening of the upper esophageal sphincter (UES) and bolus flow. When used as a rehabilitation exercise, it significantly improves the duration of the hyoid movement and the duration of the UES opening^27^.

McCullough et al.^28^ performed a research to determine if the intensive exercise using the Mendelsohn maneuver would improve swallowing physiology. The Mendelsohn maneuver, used as a rehabilitation exercise, improved the duration of the anterior and superior maxillary movement of the hyoid and the duration of the UES opening. With a similar goal, McCullough et al.^27^ stated that it seems possible that the use of the Mendelsohn maneuver as a rehabilitation exercise may have a greater impact on swallowing durations than on structural movements. Changes in the coordination of structural movements with duration measures, however, require further investigation. When the Mendelsohn maneuver was used as a compensation mechanism, duration measures also appeared to be more affected than measures of structural movements. Thus, the data reported in this research support the use of the Mendelsohn maneuver as an exercise to improve the swallowing physiology^27^.

A synthesis of the results discussed above is shown in Table 5.

**Table 5.** Interventional clinical studies using the Mendelsohn maneuver and EMG with biofeedback and acupuncture for stroke patients.

Reference	Country	Patients	Diagnosis	Study design	Outcomes	Intervention	Intervention time	Results	Follow-up
McCullough, G.H. et al., 2012 ^28^	USA	18	Stroke	Randomized	VFSS (DOHME and DOHAME)	Group A -2 weeks of treatment with the Mendelsohn maneuver and EMG with feedback and 2 weeks without treatment. Group B -2 weeks without treatment and 2 weeks with treatment.	45 min sessions, 2 times a day	DOHME and DOHMAE significantly improved (P = 0.011 and 0.009) after treatment.	No follow-up
McCullough, G.H; Kim, Y., 2013 ^27^	USA	18	Stroke	Randomized	VFSS (HME, HMAE, UES)	Group A -2 weeks of treatment with the Mendelsohn maneuver and EMG with feedback and 2 weeks without treatment. Group B -2 weeks without treatment and 2 weeks with treatment.	45 min sessions, 2 times a day	No significant improvement after treatment	1 month, 1 year
Xia, W. et al., 2015^38^	China	124	Stroke	Clinical; Randomized; Double blind	SSA; DOSS	GI-TC and acupuncture GC -TC	24 sessions/30 minutes -6 times a week	SSA and DOSS GI improvement in relation to the GC (P<0.01)	No follow-up
Mao, L. et al., 2016 ^39^	China	98	Stroke	Prospective	VFSS; SSA	GI -TC and acupuncture GC -TC	20 sessions/30 minutes -5 times a week	VFSS and SSA GI improvement in relation to the GC (P=0.007 and P=0.007)	No follow-up
Chen, L. et al., 2016 ^40^	China	250	Stroke	Randomized; Double-blind; Controlled	NIHSS; VFSS; SSA	GI -TC and acupuncture GC -TC	18 sessions/30 minutes -6 times a week	GI improvement in relation to the GC: NIHSS (p < 0.001), VFSS (p < 0.001) and SSA (p =0.037)	1, 3, 7 weeks

VFSS: Videofluoroscopy; DOHME: Duration of Hyoid Maximum Elevation; DOHAME: Duration of Hyoid Maximum Anterior Excursion HME: Hyoid Maximum Elevation; HMAE: Hyoid Maximum Anterior Excursion; UES: Upper Esophageal Sphincter; EMG: Surface Electromyography; SSA: Standardized Swallow Assessment; NIHSS: NIH Stroke Scale; DOSS: Dysphagia Outcome and Severity Scale; GI: Intervention group; GC: Control group; TC: Conventional Therapy.

### Acupuncture 

Acupuncture is a simple, inexpensive, primary medical procedure that has been widely used in China and other parts of East Asia for many years. Needles are inserted at acupuncture points to produce a "qi" response in which the patient feels pain or heaviness in the area around the needle^38^.

Xia et al.^38^ evaluated the effect of acupuncture on swallowing function in patients with dysphagia after stroke. The intervention group received standard therapy and acupuncture and the control group received only standard therapy. Although it was concluded that acupuncture combined with conventional swallowing therapy may be beneficial, the study had a significant limitation due to the lack of a control group for acupuncture alone. In addition, short-term evaluation and lack of follow-up were factors that prevented the evaluation of a long-term therapeutic effect.

Another study found that acupuncture combined with swallowing therapy can improve the swallowing function in post-stroke patients^39^. The study conducted by Mao et al.^39^ proved that acupuncture in combination with standard swallowing therapy was effective for post-stroke dysphagia, corroborating the findings presented by Xia et al.^38^. However, several limitations prevent this conclusion from being generalized, so it cannot be said that acupuncture alone is capable of providing a high level of rehabilitation.

A similar study was conducted by Chen et al.^40^. This study has shown that acupuncture is safe and has several additional effects in improving neurological deficits, swallowing disorder, cognitive impairment, and lower limb function. However, no significant improvement in the upper limb function was observed during this short-term study.

A summary of the results in the articles using acupuncture techniques is shown in Table 5.

### Cervical isometric exercises

Cervical isometric exercises to improve dysphagia and cervical spine malalignment was applied in 70 patients in a randomized controlled trial. The exercises were carried out in all 4 directions (by placing their hand or the hand of their personal assistant on their head and contracting their neck muscles under forward-backward-sideward resistance). Swallowing was improved in the experimental group compared to the control group^44^. 

## DISCUSSION

The purpose of this review was to assess recently studied therapies for dysphagia rehabilitation. Numerous studies of a wide range of interventions were included. However, they differed not only in terms of the therapy conducted, but also in terms of sample size, outcome measurement methods, intervention times and follow-up time. These differences presented a challenge to combine and summarize the results, and to compare and define which is the most effective treatment for post-stroke dysphagia. 

Considering that this neuromuscular process is complex and involves dozens of muscles and six pairs of cranial nerves, there are many symptoms that affect a dysphagic individual. Therefore, it is difficult to elaborate a single exercise protocol (in the case of conventional therapy) that will effectively improve the condition. Scientific evidence highlights the benefits of conventional therapy in improving the swallowing pattern of a dysphagic individual. However, the search for new therapeutic techniques that can increase this benefit is constant. 

Studies on the efficacy of therapeutic interventions for rehabilitation of adult patients with dysphagia after stroke are still limited. Most techniques are used in combination with conventional therapies, which makes measuring the efficacy of other techniques alone inconclusive. Among the reviewed therapies, electrotherapy, associated or not with conventional therapy, was the most frequently used. In both cases, it proved to be a method with significant results for the rehabilitation of dysphagia. Similarly, neuromodulation applied in areas such as the motor cortex of the tongue and pharynx, as mentioned in the included studies, also lead to an improvement in the swallowing pattern. Tongue pressure resistance exercises and precision training, the Shaker exercise and acupuncture also showed significant results for rehabilitation.

Neuromodulation is not a possibility in many healthcare institutions that admit patients with acute stroke, making this therapy technique difficult to access, especially for low-income patients. An advantage of the SE is that it is a non-invasive therapy, does requires no any additional cost or equipment and can be easily performed at the bedside with the assistance of a caregiver^37^. However, a limitation of the SE is that coordinated movements and resistance are required, and many patients in the acute phase of stroke do not have this capability. Pharyngeal electrical stimulation (PES) is also considered a promising treatment for dysphagia after stroke. However, the results of the studies included in this review are contrary to this. With regard to tongue pressure resistance exercise, it is important to emphasize that isometric and isotonic exercises are commonly used in conventional therapy to improve the amplitude and increase the force of tongue movements. The tongue is an essential organ for the proper functioning of the safe swallowing process.

The majority of the studies used videofluoroscopy of swallowing as the gold standard evaluation method. The method allows the swallowing dynamics to be visualized from the preparatory phase to the opening of the upper esophageal sphincter. It is also possible to identify the tracheal aspiration, laryngeal penetration, and oral and pharyngeal residues, which is important for a detailed analysis of the various changes that may occur in a dysphagia disorder of any degree. Videofluoroscopy helps in selecting the most appropriate technique and therapeutic plan to improve the swallowing pattern. Ideally, this examination should be available in all health centers admitting patients in the acute phase of stroke. 

In conclusion, this review highlights the main interventions for dysphagia of patients after stroke. Among the techniques used, conventional therapy remains the best strategy, achieving positive results alone or combined with various rehabilitation therapies. However, greater consistency between science and clinical practice is needed to allow a comparison between different techniques. Dysphagia is a potentially treatable symptom in post-stroke patients and deserves attention, and its treatment may increase patients’ quality of life. In addition, even if conventional therapy is empirically considered essential for the rehabilitation process, its effect would be strengthened by studies that scientifically support this technique.
